# The Construction of Biomimetic Cementum Through a Combination of Bioskiving and Fluorine-Containing Biomineralization

**DOI:** 10.3389/fbioe.2020.00341

**Published:** 2020-04-24

**Authors:** Tao Yang, Yanshan Li, Yubing Hong, Li Chi, Chuanzi Liu, Yu Lan, Qinmei Wang, Yingjie Yu, Qiaobing Xu, Wei Teng

**Affiliations:** ^1^Hospital of Stomatology, Guangdong Provincial Key Laboratory of Stomatology, Institute of Stomatological Research, Guanghua School of Stomatology, Sun Yat-sen University, Guangzhou, China; ^2^Laboratory of Biomaterials, Key Laboratory on Assisted Circulation, Ministry of Health, Cardiovascular Division, The First Affiliated Hospital, Sun Yat-sen University, Guangzhou, China; ^3^Institute of Translational Medicine, The First Affiliated Hospital, Shenzhen University, Health Science Center, Shenzhen, China; ^4^Department of Biomedical Engineering, Tufts University, Medford, MA, United States

**Keywords:** biomimetic cementum, bioskiving, fluorine-containing biomineralization, mineralized collagen fiber, cementogenesis

## Abstract

Despite tremendous attention is given to the construction of biomimetic cementum for regeneration of tooth cementum, the lack of recapitulating the composition and hierarchical structure of cementum often leads to the poor performance of constructed materials. How to highly mimic the sophisticated composition and hierarchy of cementum remains a longstanding challenge in constructing the biomimetic cementum. Inspired by cementum formation process, a novel construction approach via a combination of bioskiving and fluorine-containing biomineralization is developed in this study. The alternative collagen lamellae (ACL) that can highly mimic the rotated plywood structure of cementum collagen matrix is fabricated via bioskiving. Followed by biomineralization in the amorphous calcium phosphate (ACP) solution with different concentration of fluorine, a series of biomimetic cementum is constructed. Screened by physicochemical characterization, the biomimetic cementum with the composition and hierarchical structure highly similar to human cementum is selected. Through *in vitro* biological assay, this biomimetic cementum is proven to significantly promote the adhesion, proliferation, and cementogenic differentiation of periodontal ligament cells (PDLCs). Furthermore, *in vivo* study demonstrates that biomimetic cementum could induce cementogenesis. This biomimetic cementum constructed via combinatory application of bioskiving and fluorine-containing biomineralization stands as a promising candidate for achieving cementum regeneration.

## Introduction

The cementum or root cementum is a thin mineralized tissue covering the tooth root surface. As a significantly vital tissue joining the tooth to the alveolar socket, the cementum performs an irreplaceable role in protecting the root dentine from external stimulus, serving as the anchoring site for periodontal ligament (PDL) and resisting multi-directional stress during mastication (Park et al., 2017). The composition and hierarchical structure are the key issues endowing the cementum with unique biological and mechanical properties to fully exert its function. Unfortunately, the composition and hierarchical structure of cementum are extremely vulnerable under the frequently-occurring disease such as the root caries and periodontitis (Li et al., 2019). The destroyed cementum not only severely inhibits the bioactivity of PDL cells (PDLCs) and affects subsequent PDL formation, but also may initiate and aggravate root dentine resorption (Alyahya and Alqareer, 2017). Of note, the self-repairing capacity of cementum is limited and the repairing cementum is quite different from the physiological status (Chen et al., 2015). Consequently, achieving cementum regeneration is of essential importance and remains a long-standing challenge need to be addressed.

Although plenty of therapeutic approaches, including guided tissue regeneration and application of enamel matrix protein, have been explored to promote cementum regeneration, the current clinical outcomes are still far from perfect (Wei et al., 2019). Recently, mimicking the microenvironment of extracellular matrix (ECM) has been emerged as a potential strategy to induce tissue regeneration (Chen et al., 2015; Jiang et al., 2015; Wang et al., 2018; Chuang et al., 2019; Ding et al., 2019; Zhang et al., 2019). Through simulating the physicochemical cues of cementum ECM, the biological behaviors of PDLCs could be tuned and facilitated the cementogenesis (Chen et al., 2016). The composition and hierarchical structure are always the crucial parameters need to be highly simulated as for constructing the biomimetic ECM (Zhang et al., 2016; Li et al., 2017; Cui et al., 2019). As for the composition, the cementum is predominantly composed of type I collagen and fluorine-containing nano-hydroxyapatite (nFHA) (Kato et al., 1990). The content of fluorine was higher in cementum than other mineralized hard tissues (Tsuboi et al., 2014). Fluorine played a special role in cementum. Fluorine is a catalyst of biomineralization. It can accelerate the deposition of calcium ion and induce the conversion of precursors to apatite, which is be beneficial to the remineralization of cementum (Feroz and Khan, 2020). The solubility product constant of FHA (7.36 × 10^–60^) is lower than hydroxyapatite (2.35 × 10^–59^) in dilute phosphoric acid solution, thus, the high content of fluorine in cementum is favorable for resisting acid and preventing dental caries (Aoba, 1997). Moreover, with fluorine incorporated into HA, the mechanical properties of cementum including hardness, elastic modulus, and toughness were enhanced, which contributed to resisting multi-directional stress during mastication. In addition, Fluorine can stimulate cell to generate mineralized tissues *in vivo* (Harrison et al., 2004; Dawood et al., 2018). As for the hierarchical structure, directed by the specific pattern of spatial distribution, nFHA selectively deposited in collagen fiber both intrafibrillarly and extrafibrillarly, thus forming F-containing mineralized collagen fiber. F-containing mineralized collagen fiber, acting as the building block of cementum, further organizes into the complex hierarchical structure. The cementum is conventionally classified into cellular and acellular form. The cellular cementum is overwhelming structure which covers the apical and interradicular regions of root, constituting the major ECM of cementum and exerting a pivotal role in resisting and dispersing masticatory force (Yamamoto et al., 2019). Its hierarchy is characterized by unique alternating lamellar structure described as the twisted plywood model (Yamamoto et al., 2010, 2016). This sophisticated hierarchy consists of multilayers of lamellae (only a few microns thick) which are parallel to the root surface. F-containing mineralized collagen fibers are highly aligned in a given lamella and rotate from lamella to lamella (Yamamoto et al., 2010, 2016).

With the growing understanding of polymer-induced liquid-precursor process, biomineralization based on amorphous calcium phosphate (ACP) has been widely applied in synthesizing mineralized collagen fiber. As demonstrated by plenty of studies, multiple collagen fiber arrangement patterns including randomly distributed collagen fiber and highly organized collagen fibers can be biomineralized by ACP (Hu et al., 2016; Niu et al., 2017; Zhang et al., 2018). Meanwhile, through adding fluorine into ACP and synthesizing fluorine containing ACP (FACP), fluorine containing mineralized collagen fiber can be built (Saxena et al., 2018). Therefore, we proposed that collagen scaffold with specific fiber arrangement pattern could be biomineralized via FACP. Therefore, the critical step of constructing biomimetic cementum lies in obtaining the collagen fiber arrangement mimicking alternating lamellar structure. Although some progress has been made through applying the molecular crowding technique to fabricate the densely-packed collagen matrix containing some collagen lamellae (Wingender et al., 2016, 2018), its overall structure is distinct from the cementum. Thus, manufacturing complicated collagen hierarchy of cementum stands as an unsolved dilemma in constructing the biomimetic cementum.

Bioskiving, the technique combining sectioning, stacking, and rolling procedure, is a novel sectioning-based fabrication method (Alberti et al., 2014; Ghazanfari et al., 2019). Through bioskiving, decellularized tissue such as tendon could be processed into tendon-derived collagen scaffold (Dai et al., 2012; Ko et al., 2016). Since tendon is composed of type I collagen fiber with well-aligned structure, tendon-derived collagen sheet produced *via* sectioning process is characterized by highly parallel fibers. Notably, the multi-sheeted construct can be further built through stacking sheets on top of each other and reorienting adjacent sheets at the desired angle to tune the mechanical property of the constructs (Alberti et al., 2015). Interestingly, the hierarchical structure of this construct presents somewhat similarity to the alternating lamellar structure in cementum (Reznikov et al., 2014). Both of them present the similarity including the multi-layered well-aligned collagen fibers in each layer and periodically rotated among adjacent layers. Unfortunately, the sheet obtained by bioskiving was 50 microns thick at present, which is significantly thicker than cementum lamella and may affect the seamless integration with surrounding cementum lamella. In theory, the thickness of tendon-derived collagen sheet can be precisely reduced to only a few microns during sectioning procedure. Thus, we hypothesize that the collagen hierarchy of cementum could be constructed *via* bioskiving.

**FIGURE 1:**
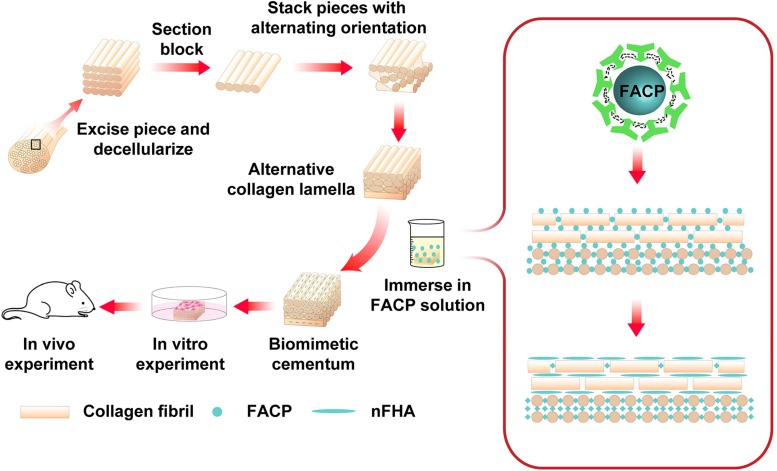
Schematic illustration of the construction of biomimetic cementum *via* a combination of bioskiving and fluorine-containing biomineralization.

The present study describes our attempt to fabricate the biomimetic cementum highly resembling the complex composition and hierarchical structure of cementum (Figure 1). FACP was prepared *via* adding different concentration of fluorine into carboxymethyl chitosan (CMCS) stabilized FACP solution. The alternating lamellar structure of collagen fiber was produced *via* bioskiving and subsequently biomineralized in the different FACP solution to construct biomimetic cementum. The human cementum was employed as the positive control. Through investigating the physicochemical properties (microstructure, component element, crystallinity, and inorganic content), the biomimetic cementum possessed the closest parameters regarding composition and hierarchical structure to human cementum was selected as optimum and its bioactivity on PDLCs was evaluated. Furthermore, the function of promoting cementum regeneration was investigated *in vivo*. To the best of our knowledge, this is the first time that both composition and hierarchical structure of cementum could be highly imitated, which offers the favorable microenvironment for cementum regeneration. Employing this biomimetic cementum as the fundamental structure, the cementum-PDL-alveolar bone complex could be constructed. This novel fabrication approach combining the bioskiving and fluorine-containing biomineralization could provide a promising approach to facilitate periodontal regeneration.

## Materials and Methods

### Synthesis and Characterization of FACP

A series of 1.2 g CMCS (Xiya Reagent Co., Ltd. China) were completely dissolved in 100 ml Tris buffer containing 14.46 mM K_2_HPO_4_ and different masses of NaF. Subsequently, 20 ml CaCl_2_ (23.79 mM) was added dropwisely. The final fluorine concentration was 5 mM, 2.5 mM, 1.25 mM, and 0.625 mM. During the synthesis of FACP, the pH of FACP solution was precisely control to 8.0 regulated by HCl and the temperature was kept at 25°C *via* the thermostat. FACP was named according to the fluorine concentration. After 10 min stirring, the solution was collected and filtrated through a polytetrafluoroethylene (PTFE) membrane with 0.1 μm pore size, followed by washing with deionized water and ethanol, freeze drying, and grounding into powders. The fluorine containing calcium phosphate without CMCS was synthesized in the same way and set as the control.

To observe the morphology, the resulting products were mounted on stub, sputter coated with gold particles, and observed using a scanning electron microscopy (SEM, Quanta 400F) at an accelerating voltage of 20 kV. Chemical element was analyzed by energy dispersive spectrum (EDS). The powders were further characterized by transmission electron microscopy (TEM, FEI Tecnai G2 Spirit) at an accelerating voltage of 120 kV. Specimens were prepared by dispersing in ethanol and dropping onto a 300 mesh copper grids coated with an amorphous carbon film. Selected area electron diffraction (SAED) was used to identify the crystallinity of minerals. Meanwhile, the crystal structure was investigated by X-ray diffractometer (XRD, Empyrean), which was performed at 40 kV and 35 mA. The data were collected in the 2θ range of 10–60° with a step size of 0.02°. The powders were also tested by Fourier transform infrared (FTIR) spectroscopy (Nicollet 6700). Spectrum was collected ranging from 400 to 2000 cm^–1^. To examine the stability of FACP in solution, fresh FACP solution was added into 96 well plate and the optical density (OD) at a wavelength of 650 nm was examined every day for up to 7 days by the Plate Reader (Beckman Coulter AD 340). The mineral content of FACP was determined by thermogravimetric analysis (TGA, TG209F1 libra) in an air atmosphere at a heating rate of 10°C/min up to 900°C. Fluorine content of the mineral (the ash from TGA) was investigated (Saxena et al., 2018). Briefly, a known mass of sample (2–4 mg) was dissolved in 1 ml of 0.5 M perchloric acid for 1 h and then added 4 ml of 0.5 M trisodium citrate. Fluorine content was determined by a fluorine selective electrode (Orion 9609BNWP with Orion pH/ISE meter 710) and series of diluted standard fluorine solutions within a range of 1–100 ppm. To test the phase transformation, the FACP was collected from solution after 4 days and evaluated through SEM, TEM, SADE, XRD, and FTIR.

### Construction and Characterization of Alternative Collagen Lamellae (ACL) Fabricated via Bioskiving

Collagen section was fabricated according to the previous method with modification (Alberti et al., 2015). Briefly, bovine Achilles tendons were cut into blocks and decellularized in a 1 mM Tris buffer containing 1% w/v sodium dodecyl sulfate and 0.1 mM ethylene diamine tetraacetic acid. All the reagent mentioned above were obtained from Sigma (Sigma Chemical Co., United States). The tendon blocks were kept shaken (150 r/min) for 3 days and the buffer was refreshed every day. Following washed with plenty of deionized water, the tendon blocks were immersed in DNAse I solution (1 mg/ml) for 1 day. After thorough rinse with deionized water, the blocks were embedded in OCT (Sakura, United States) compound and sectioned *via* the cryomicrotome (CM1950). The thickness of each collagen section was controlled at 10 μm. Before the stacking procedure, the collagen sections were immersed in deionized water to fully remove OCT compound and dehydrated in gradient ethanol solution (25, 50, 75, and 100%). After dehydration, the collagen sections became hardened and their operability was improved. Then, collagen section was stacked on a PTFE block. Following the first collagen section, each subsequent collagen section was stacked with the fiber orientation periodically rotated by 30° between adjacent layers. To prevent shrinkage of collagen sections caused by excessive dryness in the air, the stacking procedure was conducted in the 100% ethanol solution. After stacking, the surface of alternative collagen lamellae (ACL) was covered and fixed by another PTFE block prior to immersed in deionized water for 10 min. Subsequently, the ACL fixed by PTFE blocks was taken out and dried in the air for 48 h. The stacked collagen sections were crosslinked by the hydrogen bond (Alberti et al., 2015).

Scanning electron microscopy was employed to investigate the hierarchy of ACL. Both the surface and longitudinal plane were investigated. The surface of ACL was tested by directly mounting ACL on stub. The longitudinal view of ACL was observed by embedding ACL in OCT and sectioning ACL in the direction vertical to the lamellae prior to mounting on stub.

### Fabrication and Characterization of Biomimetic Cementum

Alternative collagen lamellae was immersed in fresh prepared FACP dispersion with different fluorine concentration. The FACP dispersion was changed for every 3 days. After 12 days, the mineralized lamellae were taken out and rinsed with deionized water to remove the loosely attach minerals. The biomimetic cementum was denominated according to the fluorine concentration of FACP where ACL was immersed. The biomimetic cementum was freeze-dried prior to the characterization of TEM, SAED, SEM, EDS line scanning, XRD, FTIR, TGA, and fluorine selective electrode. The above results were compared with those of human cementum to evaluate the simulation performance. Healthy premolars aged from 20 to 30 years were collected under the approval of the Medical Ethics Committee of Hospital of Stomatology, Sun Yat-sen University (No. KQEC-2019-38, Approval date: 21 October 2019). PDLs were completely removed from tooth root by scaling. The apical third part of the root was collected and the superficial cementum was sectioned into 1 mm thickness prior to mounting on stub for SEM investigation. As for TEM, SADE, FTIR, XRD, and TGA test, samples were prepared by pulverizing cementum in liquid nitrogen and freeze-drying. The biomimetic cementum with most comparable performance with human cementum was selected for the subsequent biological assay.

### *In vitro* Biological Assay

Human PDLCs were collected as described previously (Dan et al., 2014). Briefly, PDL tissues were minced from the root of the extracted healthy premolars and digested with collagenase (3 mg/ml) for 30 min at 37°C. Single cell were isolated through a 70 mm strainer. The PDLCs were incubated in αMEM (Gibco, United States) supplemented with 10% FBS (Gibco, United States) and 100 U/ml penicillin/streptomycin (Gibco, United States) in humidified atmosphere at 37°C and 5% CO_2_. The culture medium was changed every 2 days and cell passage was performed at a ratio of 1:3 when the cells reached approximately 90% confluence. PDLCs from passages 3 to 5 were employed in this study.

Prior to the testing, the biomimetic cementum was cut into approximately 4 mm × 4 mm before placed in 96 well plate and 6 mm × 6 mm in 48 well plate. The biomimetic cementum was sterilized by 75% ethanol and thoroughly washed with PBS. The decellularized human cementum was processed in the same method and set as a positive control. The ACL was set as the negative control.

### Cell Attachment and Proliferation Assay

The cell attachment assay under various concentrations of FBS was conducted based on previously described method (Lee et al., 2009; Wang et al., 2017). PDLCs of passage 3–5 were collected and further washed by PBS three times to remove remaining FBS in culture medium. A series of 100 μl culture media containing various concentration of FBS (0.5, 1, 5, and 10%) were prepared and 1 × 10^4^ PDLCs was added prior to seeding on the surface of ACL, biomimetic cementum, or cementum placed in 96 well plate. After incubating at 37°C for 12 h, the culture medium was aspirated out and the PDLCs-laden specimens were gently rinsed with 100 μl PBS for three times. Both the culture medium and rinsed PBS were collected and mixed, which contained the non-adherent as well as loosely attached PDLCs. The mixed solution was blown evenly and 20 μl was taken to measure the cell number by the cell counter (Cellometer Auto 1000). During the cell counting, three fields were randomly selected and measured. Three samples were taken from each well and examined during cell attachment assay. The cell attachment assay was repeated three times. Cell attachment efficiency was calculated *via* the following equation:

$$\mathrm{cell}\mathrm{attachment}\mathrm{efficiency}\left(\%\right)=\frac{n_{0}-n_{1}}{n_{0}}\times 100\%$$

*n*_0_ indicated the number of initial seeded cells and *n*_1_ indicated the number of non-adherent and loosely attached cells.

As for cell proliferation assay, PDLCs were seeded on the surface of ACL, biomimetic cementum, or cementum placed in 96 well plate at a density of 5 × 10^3^ cells per specimen with 200 μl culture medium in each well and three wells of each group were set. The culture medium was refreshed every 2 days. The proliferation was evaluated by CCK-8 assay kit (Dojindo, Japan) as manufacture instruction. At 1, 4, and 7 days, the culture media were aspirated out and specimens seeded with PDLCs were gently washed three times with PBS. Then, 180 μl of fresh culture medium containing 20 μl of CCK-8 reagent was added in each well. After incubating at 37°C for 2 h, 180 μl medium was taken out and centrifuged at 500 r/min. 150 μl supernatant was taken out and its OD value at 450 nm was measured by the Plate Reader. Each experiment was independently repeated triplicately. CCK-8 reagent can be reduced by dehydrogenase in cell to generate the yellow-color formazan dye, which can be dissolved in culture media and directly proportional to the number living cells. Therefore, the OD value was employed in this study to evaluate the cell proliferation.

### Cementogenic Differentiation Assay

5 × 10^4^ PDLCs were seeded on each specimen placed in 48 well plate for further examination. 24 h after seeding, the media were changed into differentiation media containing 10 mM β-glycerophosphate, 100 μM ascorbic acid, and 10 nM hexadecadrol based on the previous methods (Flores et al., 2008; Zhao et al., 2013). Differentiation medium was refreshed every 3 days. Quantitative real-time polymerase chain reaction (RT-qPCR) was used to measure mRNA expression of PDLCs. After culturing 7 and 14 days, total RNA of PDLCs-laden on specimens was harvested with RNA Quick Purification kit (ESscience, China). The concentration of RNA was measured and the purity of RNA was determined by the absorbance at 230, 260, and 280 nm using a NanoDrop 2000/2000c Spectrophotometers. The 260/280 values between 1.8 and 2.0 and 260/230 values between 1.8 and 2.2 suggested that the RNA was free of contamination. 1 μg RNA was reverse-transcribed to cDNA using the PrimeScript RT Master Mix (Takara, Japan). cDNA was used for further amplification and RT-qPCR reactions were performed using LightCycler 480 SYBR Green I Master (Roche Diagnostics, United States) according to the manufacturer’s instruction. The master mix (20 μl) per well contained 10 μl SYBR green, 3 μl water, 2 μl diluted (1:10) primers (F+R), and 5 μl diluted (1:10) cDNA and was replicated in three wells of a 96-well plate. Sequence of primers is listed in Table 1. The reactions were set as the following cycling conditions: 95°C for 5 min, 40 cycles at 95°C for 10 s, 60°C for 20 s, 72°C for 20 s, and a melting curve from 60 to 95°C for increments of 0.5°C for 15 s. Relative expression of target genes normalized by GADPH was calculated by the 2^–ΔΔ*Ct*^ method. Three wells per specimen were examined in three independent experiments.

**TABLE 1:** The primers for RT-PCR.

Gene		Primer (5′→3′)
Cementum protein 1 (*CEMP1*)	F	ACCAAGAGTGCTTCCCCCACAC
	R	CCCATGTGGCAAACACGGGC
Bone sialoprotein (*BSP*)	F	GATTTCCAGTTCAGGGCAGTAG
	R	CCCAGTGTTGTAGCAGAAAGTG
Alkaline phosphatase (*ALP*)	F	TTGTACGTCTTGGAGAGGGC
	R	TCAGAAGCTCAACACCAACG
Osteopontin (*OPN*)	F	CTCCATTGACTCGAACGACTC
	R	CAGGTCTGCGAAACTTCTTAGAT
Osteocalcin (*OCN*)	F	CACTCCTCGCCCTATTGGC
	R	CCCTCCTGCTTGGACACAAAG
*GADPH*	F	TCAGCAATGCCTCCTGCAC
	R	TCTGGGTGGCAGTGATGG

The immunostaining of cementogenic differentiation marker, CEMP1, was tested at 7 and 14 days, the specimens laden with PDLCs were washed twice with PBS and fixed in 4% paraformaldehyde for 30 min. After washing with PBS, cells were treated with 0.1% Triton X-100 for 20 min and blocked with 1% BSA for 1 h. Then, cells were incubated with rabbit antitype CEMP1 antibodies (1: 100 dilution, Abcam, United Kingdom) overnight at 4°C. Subsequently, cells were incubated with FITC-conjugated goat antirabbit IgG (1: 300 dilution, Affinity Biosciences, United States) for 1 h at 25°C. Nuclei were stained with DAPI (Gen-view Scientific Inc., United States). The images were captured by confocal laser scanning microscope (CLSM, Olympus).

### *In vivo* Biological Assay

Nine CB-17 SCID male 6-week-old nude mice were purchased from Sun Yat-sen University Experimental Center for Animal Experiment. All procedures were performed in accordance with an approved animal protocol (Institutional Animal Care and Use Committee, Sun Yat-sen University (No. SYSU-IACUC-2019-000326). Human PDLCs at passage 3 were seeded on the surface of biomimetic cementum (5 mm × 5 mm) in 48 well plate at a concentration of 1 × 10^5^ cells per well and cultured in αMEM media supplemented with 10% FBS and 100 U/ml penicillin/streptomycin. After culturing for 7 days, the biomimetic cementum laden with PDLCs was implanted into subcutaneous pockets of mice (*n* = 3) (Kim et al., 2012). The mice were anesthetized by intraperitoneal injection of 2% sodium pentobarbital (40 mg/kg), the dorsal skin was incised, and biomimetic cementum laden with PDLCs was placed on the muscle surface prior to suturing. The ACL seeded with PDLCs and biomimetic cementum without PDLCs were also implanted and set as the control group. Two weeks later, all the mice were sacrificed *via* overdose anesthesia. The materials with their surrounding tissues were harvested, fixed in 4% paraformaldehyde overnight, and dehydrated in a graded ethanol series. Then, embedded in paraffin and then sectioned in 5 μm for further HE and Masson trichrome staining. In addition, immunohistochemistry staining was conducted. The sections were quenched in 3% H_2_O_2_ for 20 min, blocked in 3% BSA for 30 min, and then incubated with rabbit antitype CEMP1 antibodies (1: 100 dilution). Goat antirabbit IgG (Servicebio, China) at a dilution of 1:200 was further incubation for 30 min at room temperature, followed by treatment with diaminobenzidine solution (Servicebio, China). The slides were scanned and observed by Aperio Digital Pathology (Leica).

### Statistical Analysis

All the experiments were repeated triplicately unless otherwise stated. Data are expressed as mean ± standard deviation. Differences between groups were assessed by one-way analysis of variance (ANOVA) and a *post hoc* Bonferroni *t*-test. Significance was set at *p* < 0.05.

## Results

### Synthesis and Characterization of FACP

**FIGURE 2:**
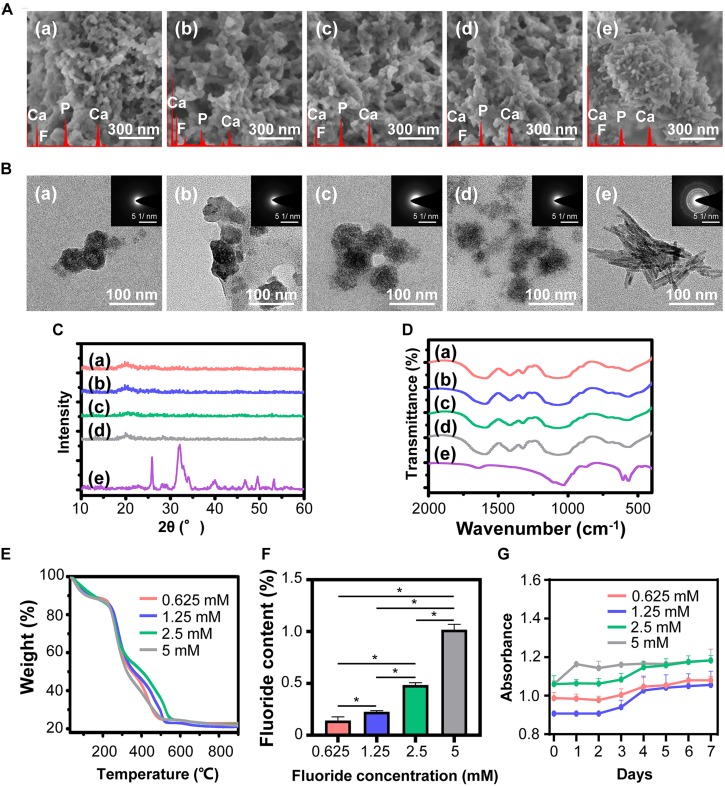
Synthesis and characterization of FACP with different fluorine concentration. **(A)** SEM images, **(B)** TEM images and the corresponding SAED pattern, **(C)** XRD spectra, and **(D)** FITR spectra of FACP with different fluorine concentration. In **A**–**D**, the initial fluorine concentration of FACP was **(a)** 0.625, **(b)** 1.25, **(c)** 2.5, and **(d)** 5 mM. **(e)** Synthesized fluorine containing calcium phosphate without CMCS. **(E)** TGA result and **(F)** fluorine content of FACP. **(G)** OD profiles (650 nm) of FACP dispersion up to 7 days. Asterisk on top of each bar indicated statistically difference between groups (*p* < 0.05).

Characterization of fluorine containing calcium phosphate is shown in Figure 2. Evenly distributed aggregates containing calcium, phosphorus and fluorine element with diameter around 50 nm were detected in the SEM images of FACP [Figures 2A(a–d)]. TEM images revealed that these aggregates were composed of numerous nanoparticles featured by no dots or ring pattern in SAED [Figures 2B(a–d)], demonstrating their amorphous phase. On the contrary, the SEM and TEM images exhibited the needle shaped structure in fluorine containing calcium phosphate without CMCS [Figures 2A(e), B(e)]. The corresponding SAED revealed characteristic discrete ring patterns ascribing to the crystalline phase. The typical crystal diffraction peaks of FHA around 32.2°, 31.7°, 32.8°, and 25.8°, ascribing to (112), (211), (300), and (002) plane, were detected in the XRD spectra (Figure 2C). With the addition of CMCS, only the amorphous diffraction peak (20°–30°) was observed. A single halo peak (around 548 cm^–1^) associated with *ν*4 PO_4_^3–^ bending mode was found in FTIR spectra of FACP [Figures 2D(a–d)], revealing the presence of the amorphous phase. This single halo peak was split into two peaks (around 529 and 567 cm^–1^) in the absence of CMCS, which was related with the crystalline phase of FHA [Figure 2D(e)]. The above results indicated that FACP was successfully constructed with the addition of CMCS. The mass content of inorganics in FACP was around 25 % (Figure 2E) and no significant difference of inorganic mass existed among different FACP groups (Figure 2F). The fluorine content was 0.14, 0.22, 0.48, and 1.02% in 0.625, 1.25, 2.5, and 5 mM group of FACP, respectively. The fluorine content was close to the theoretical adding amount of fluorine, implying that the fluorine was fully incorporated into the FACP. Since the stability of FACP was crucial in biomineralization procedure, the OD value reflecting FACP stability was examined (Figure 2G). The OD value remained stable for the first 3 days and started to increase afterward in 0.625, 1.25, and 2.5 mM group, indicating the excellent stability of FACP. However, the OD value of 5 mM group remarkably increased at 1 day, which may be associated with the quick phase transformation induced by the high fluorine concentration.

Scanning electron microscopy images (Supplementary Figure S1A) showed that transformed FACP exhibited the finger-like morphology instead of evenly distributed aggregates, which was characterized by the nano-sized needle shaped under TEM observation (Supplementary Figure S1B). The corresponding SAED revealed characteristic ring patterns of crystalline phase. The typical peaks (32.2°, 31.7°, 32.8°, and 25.8°) of FHA were detected in XRD spectra (Supplementary Figure S1C) and the split peaks (around 529 and 567 cm^–1^) were also found in FTIR spectra (Supplementary Figure S1D), suggesting that FACP transformed to nFHA within 4 days and could be applied to construct the F-containing mineralized collagen fibers.

### Fabrication of ACL and Biomimetic Cementum

**FIGURE 3:**
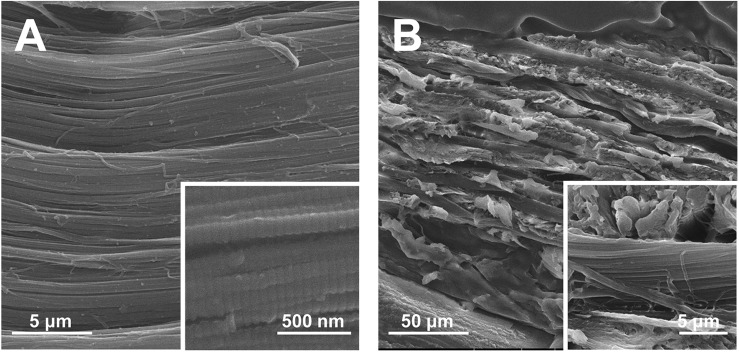
SEM images of ACL. **(A)** The surface and **(B)** longitudinal section view.

The hierarchical structure of ACL was examined by SEM. In the surface view (Figure 3A), bundles of collagen fibers with striated nanostructure were highly aligned. In the longitudinal view (Figure 3B), multiple layers of collagen fiber lamellae were stacked. The thickness of collagen fiber lamella was approximately 10 μm and the orientation of fiber rotated among lamellae.

**FIGURE 4:**
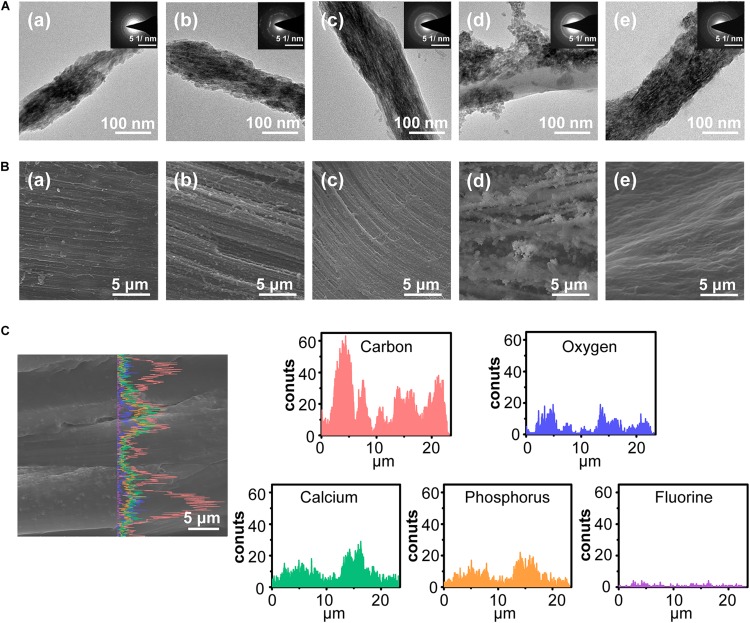
The morphology of biomimetic cementum. **(A)** TEM images and the corresponding SAED pattern of biomimetic cementum. **(B)** The surface view of SEM image. **(a–d)** indicated 0.625, 1.25, 2.5, and 5 mM group of biomimetic cementum, respectively. **(e)** Cementum. **(C)** The SEM images with corresponding EDS line scanning along the longitudinal section of 2.5 mM biomimetic cementum.

After immersing ACL in different FACP solution, a series of biomimetic cementum were constructed. As the fluorine concentration of FACP increased from 0.625 to 2.5 mM, both intrafibrillar and extrafibrillar mineralization of collagen fibers were gradually enhanced (Figure 4A). Of note, the structure of F-containing mineralized collagen fiber in 2.5 mM biomimetic cementum group was highly comparable to that in human cementum. However, as the fluorine concentration of FACP reached 5 mM, the mineral was randomly deposited on the extrafibrillar surface and the intrafibrillar mineral was almost disappeared. The SEM images (Figure 4B) of biomimetic cementum exhibited a similar trend. The roughness was gradually heightened from 0.625 to 2.5 mM group. The 2.5 mM group of biomimetic cementum showcased the highly structural similarity with human cementum featured by well-aligned mineralized collagen fibers. Notably, large crystals were unevenly distributed in 5 mM group of biomimetic cementum and the parallel pattern of fibers was obscure. The EDS line scanning along the mid-third longitudinal section of 2.5 mM biomimetic cementum is shown in Figure 4C. Besides O and C which were the basic element of collagen fibers, abundant Ca, P, and F were detected, indicating that FACP efficiently penetrated into the ACL and F-containing mineralized collagen fibers were generated.

**FIGURE 5:**
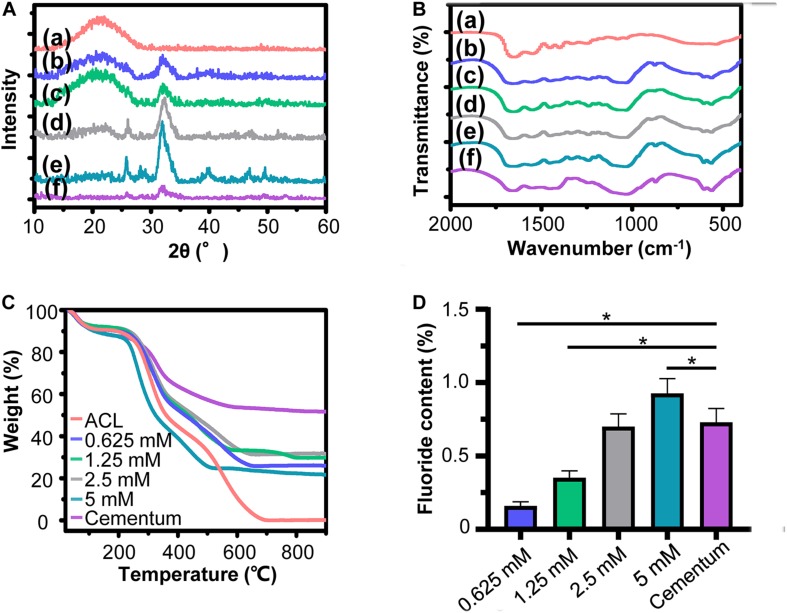
Characterization of biomimetic cementum. **(A)** XRD spectra, **(B)** FITR spectra, **(C)** TGA result, and **(D)** fluorine content. In **A, B**, **(a)** ACL, **(b–e)** indicates 0.625, 1.25, 2.5, and 5 mM group of biomimetic cementum, respectively. **(f)** Cementum. Asterisk on top of each bar indicated statistically difference between groups (*p* < 0.05).

The XRD spectra of biomimetic cementum exhibited the typical peak of FHA from 32.2°, 31.7°, and 32.8°, which were also detected in the human cementum (Figure 5A). Meanwhile, the biomimetic cementum possessed the similar FTIR spectrum with human cementum (Figure 5B). The peaks at 560 and 602 cm^–1^ were ascribed with the stretching modes of *υ*4 and *υ*2 O-P-O bending modes in FHA. The peaks located at 1650–1635, 1550–1535, and 1240 cm^–1^ were attributed to the amide I, amide II, and amide III of collagen, respectively. Shown in Figure 5C, although the mineral content in biomimetic cementum was lower than that of human cementum, approximately 23–35% of inorganic mass could still be obtained (Figure 5C). The mineral content increased from 0.625 to 2.5 mM group biomimetic cementum (28.3, 32.1, and 34.0%). However, the mineral content reduced to 23.8% in 5 mM group, which may be related with the reduction of intrafibrillar mineralization. Shown in Figure 5D, the fluorine content increased from 0.625 to 5 mM group, and there was no statistical difference between 2.5 mM group and human cementum. Based on the above results (Figures 4, 5), the 2.5 mM group has the closest physicochemical properties to the human cementum and therefore was selected as the optimum biomimetic cementum to conduct the biological experiments.

### *In vitro* Experiment

**FIGURE 6:**
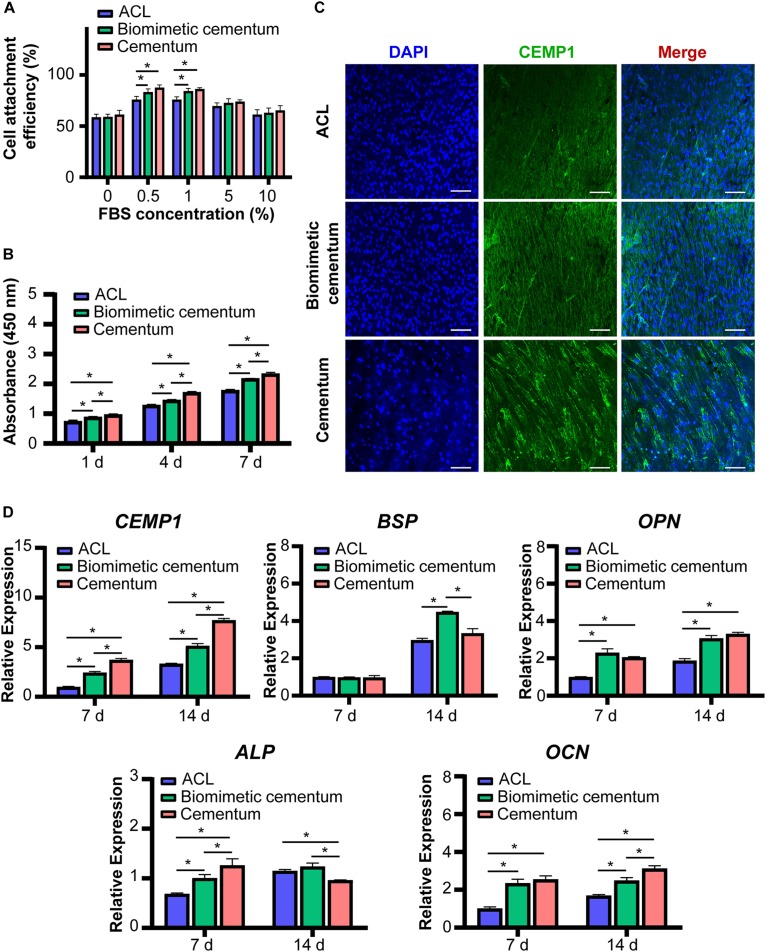
The *in vitro* effect of biomimetic cementum on the PDLCs. **(A)** Cells adhesion under various concentrations of FBS. **(B)** Proliferation. **(C)** The immunofluorescent image of CEMP1 protein expressed in PDLCs after 14 days. Blue fluorescence represents nuclei and green fluorescence represents CEMP1. Scale bar: 100 μm. **(D)** Gene expression results of *CEMP1*, *ALP*, *BSP*, *OPN*, and *OCN*. Asterisk on top of each bar indicated statistically difference between groups (*p* < 0.05).

The results of cell adhesion assay are shown in Figure 6A. In the 10 and 5% FBS environment, no statistical difference existed in cell adhesion among three materials. However, as the FBS concentration decreased to 1 and 0.5%, the biomimetic cementum significantly promoted PDLCs adhesion than ACL, and exhibited a similar cell attachment efficiency with human cementum. As reported by previous papers (van Wachem et al., 1987; Wang et al., 2017), high concentration of FBS can inhibit cell to express adhesion related proteins, thus the capacity of biomimetic cementum to facilitate cell attachment may be concealed at 10 and 5% FBS environment. Notably, as the concentration of FBS reached 0%, no serum protein adhered to the surface of the material and the cells can only secrete ECM to achieve attachment (Meng et al., 2015), which resulted in the reduced adhesion efficiency. No statistical difference was detected among three materials. The result of cell proliferation showed that the biomimetic cementum exhibited a potent ability to promote cell growth than ACL at 1, 4, and 7 days. As for the cementogenic differentiation (Figure 6D), the biomimetic cementum significantly upregulated the expression of *CEMP1*, the cementum-specific protein which only expressed in cementoblasts and its progenitor cells (Komaki et al., 2012), than ACL. *BSP* and *OPN* were two major mineralization related proteins expressed in cementum (Arzate et al., 2015). For *BSP*, no expression difference was detected among three groups at 7 days. *BSP* was significantly upregulated in biomimetic cementum at 14 days compared with other two groups. As for *OPN*, there was no statistical difference existed between biomimetic cementum and human cementum at both 7 and 14 days, which was significantly higher than ACL. A similar trend could be detected in *ALP* and *OCN* expression. Both biomimetic cementum and human cementum exhibited an upregulation expression than ACL at 7 and 14 days.

Immunofluorescence images showed expression of CEMP1 at 7 (Supplementary Figure S2) and 14 days (Figure 6C). CEMP1 was highly aligned in three groups, indicating that growth and migration of PDLCs were directed by the fiber orientation of materials. The fluorescence intensity was significantly enhanced and more CEMP1 was expressed at 14 days. Consisted with the qPCR results, the upregulated expression of CEMP1 could be found in biomimetic cementum compared with ACL.

### *In vivo* Experiment

**FIGURE 7:**
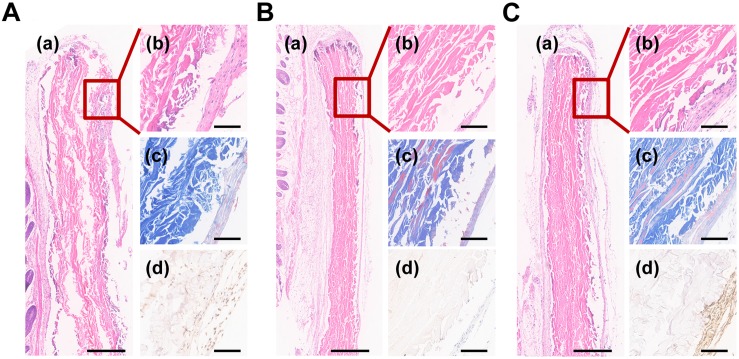
Histological images of **(A)** ACL and **(B)** biomimetic cementum without PDLCs and **(C)** biomimetic cementum seeded with PDLCs. **(a,b)** indicated the HE staining, **(c)** indicated Masson trichrome staining, and **(d)** indicated immunohistochemical staining of CEMP1. Scale bar in **a** of **A**–**C:** 500 μm. Scale bar in **b**–**d** of **A**–**C:** 100 μm.

To assess the effect to facilitate cementum regeneration, biomimetic cementum and ACL seeded with PDLCs as well as biomimetic cementum without PDLCs were implanted subcutaneously into the nude mice. After 2 weeks, both ACL and biomimetic cementum exhibited excellent tissue integration and no inflammation reaction was detected surrounding the implant (Figure 7). As for ACL, a loosely packed collagen lamellae could be observed and plenty of fissure was detected between adjacent lamellae. Only a few PDLCs were left and limited CEMP1 was expressed in ACL surface, which may be ascribed with the premature degradation of ACL (Figure 7A). As for the biomimetic cementum, a densely packed matrix and the alternating rotation of lamellae could be clearly observed (Figure 7B). No expressed CEMP1 was detected. The stable structure can also be found in biomimetic cementum laden with PDLCs (Figure 7C). Notably, its thickness was higher than that of biomimetic cementum without cell, and CEMP1 was remarkably expressed.

## Discussion

As a thin hard tissue covering the root surface and anchoring the PDL, cementum is highly vulnerable and destroyed cementum could in turn deteriorate the progress of periodontitis and root caries (Grzesik and Narayanan, 2002). For tissue regeneration, there are currently various cell-free drug strategies to achieve regeneration, such as mimicking the ECM and applying therapeutic extracellular vesicles (Gyorgy et al., 2015). Numerous studies have focused on simulating cementum ECM to promote cementum regeneration. In the field of construction of bioinspired ECM (Chen et al., 2015; Mao et al., 2015; Zhang et al., 2019), composition and hierarchical structure are the pivotal need to be simulated. As for the composition, the cementum contained abundant fluorine, which played a special role in cementum and was absent in most of constructed cementum ECM. For the hierarchy, cementum is characterized by a unique alternating lamellar structure, which was difficult to be mimicked. Referring to the formation process of cementum is a feasible avenue to address the intractable puzzle of mimicking composition and hierarchical structure. During cementogenesis (Yamamoto et al., 2016), the collagen is first secreted by cementoblast and further assembles into the collagen fiber matrix with complex hierarchy under the cell control. Then, the fluorine containing mineral was deposited in the collagen matrix and thus forming the cementum. The above process involves two critical steps: generating the sophisticated hierarchical structure of cementum collagen matrix and implementing fluorine containing biomineralization.

The hierarchy of cementum collagen matrix is intricate, which could be described by the twisted plywood model (Yamamoto et al., 2010, 2016, 2010). Demonstrated by our preliminary results (unpublished data), the ACL structure which was highly similar to the twisted plywood model can be fabricated *via* bioskiving. Shown in SEM images (Figure 3), the resulting ACL was composed by layers of collagen lamellae stacked in alternating manner. In each lamella, collagen fibers were featured by both the striated nanostructure of collagen fibers and parallelly aligned microstructure. Through precisely controlling the thickness and rotation orientation of each lamella, a highly biomimetic collagen matrix of cementum can be fabricated by bioskiving. Based on the superiorities of bioskiving, in the future, theoretically, through pre-scanning the root defect area, a customized biomimetic cementum could be fabricated with its morphology exactly fitting the defect and its thickness as well as lamella orientation highly matchable to the surrounding healthy cementum.

The other dilemma in constructing biomimetic cementum is to achieve fluorine containing biomineralization of ACL. Although employing ACP has been proven to be an effective tactic to accomplish mineralization of collagen fiber, adding fluorine into ACP could significantly affect the biomineralization procedure (Saxena et al., 2018). A series of FACP with different fluorine concentration were first prepared in our study. With the help of CMCS, the synthesized fluorine containing calcium phosphates were evidenced to be non-crystal (Figures 2A–D), remained stable in solution up to 3 days and transformed into nFHA thereafter (Supplementary Figure S1). Then, ACLs were immersed in these FACP solutions. The resulting biomimetic cementum differed remarkably. With the fluorine concentration in FACP increased from 0.625 to 2.5 mM, the inorganic mass of biomimetic cementum gradually increased (Figure 5C). When the fluorine concentration in FACP rose to 5 mM, the inorganic mass did not further augment. Meanwhile, the microstructure of biomimetic cementum exhibited a similar trend. From 0.625 mM group to 2.5 mM group, the surface roughness of biomimetic cementum increased and both intrafibrillar as well as extrafibrillar mineralization were enhanced (Figures 4A,B). The morphology of 2.5 mM group was highly similar to human cementum. However, the intrafibrillar mineralization was significantly reduced in 5 mM group, and paralleled aligned microstructure was almost disappeared due to the coverage of large, irregular agglomerates. Based on the previous research (Saxena et al., 2018) and our results, fluorine concentration may exert a dual effect on biomineralization of collagen fibers, and a threshold for fluorine concentration in FACP was supposed to exist. Below the threshold, increasing fluorine content could promote both intrafibrillar and extrafibrillar mineralization. However, as the fluorine concentration above the threshold, the physical property of FACP may change. Thus, FACP could not penetrate the intrafibrillar compartment of collagen fiber and only extrafibrillar mineralization was generated.

The biological performance is the core parameter to evaluate the simulation of cementum microenvironment. The cell adhesion was the critical process orchestrating the subsequent (Han et al., 2019; Cui et al., 2020). The biomimetic cementum exhibited a similar PDLCs attachment with human cementum (Figure 6). As for the proliferation and differentiation, the biomimetic cementum significantly promoted the cell growth and induced the cementogenic differentiation compared with ACL. Moreover, the thickness of the implanted materials remarkably increased after embedding in mice for 2 weeks. We believed that the increased thickness of ACL and biomimetic cementum may be attributed to distinct reasons. For ACL, the decellularized tendon was extremely dried before sectioning and the thickness of dry collagen lamina was 10 μm. During stacking procedure, the dried collagen laminae were stacked with the fiber orientation periodically rotated by 30° between adjacent laminae. Stacked collagen laminae were rehydrated and then dried at the room temperature. ACL was crosslinked by the hydrogen bond (Fu et al., 2015). The thickness of dry ACL was 180 μm. When ACL was embedded in mice, the ACL was rehydrated and swollen, the thickness was somewhat increased. More importantly, since the hydrogen bond was not strong enough, the stacked collagen laminae were detached and plenty of fissures were detected between adjacent lamellae (Alberti and Xu, 2016). Thus, the increased thickness was mainly associated with the loosely packed structure of ACL. However, as for the biomimetic cementum, due to the structure reinforcement of fluorine containing biomineralization, the biomimetic cementum was more stable (Gholipourmalekabadi et al., 2015), which may provide a more favorable environment for PDLCs and cells surrounding the biomimetic cementum to produce the ECM. Compared with the biomimetic cementum without PDLCs (Figure 7), those with cells seeding was much thicker and we believed that the increased thickness of biomimetic cementum was mainly attributed to the newly formed ECM. Based on the above results, through highly mimicking composition and hierarchical structure of cementum, biomimetic cementum could efficiently regulate biobehavior of PDLCs *via* providing multiple physicochemical cues.

In recognition of one of the major challenges in periodontal tissue engineering, i.e., how to fabricate the cementum with complex composition and hierarchical structure to achieve cementum regeneration, the present study verified the possibility of constructing biomimetic cementum *via* combined application of bioskiving and fluorine-containing biomineralization. This biomimetic cementum offers prodigious potential to be a competitive candidate in promoting cementogenesis. In addition, through bonding biomimetic cementum to exposed dentin *via* bioadhesive agent or dental adhesive in the future, biomimetic cementum could firmly contact with dentin and thus preventing external stimulus and dentine sensitivity. In this study, compared with human cementum, however, there is still a small gap in regenerative performance between our biomimetic material and native tooth. We believe this gap was associated with the lacking of biological cues for cell. Inspired by the previous methods (Hu et al., 2017; Li et al., 2019; Wang et al., 2019), extracellular vesicles generated from cementum related cells were supposed to load inside the biomimetic cementum, which could significantly improve the cementogenic performance.

## Conclusion

The combination of bioskiving and fluorine-containing biomineralization enables the construction of biomimetic cementum. Through highly mimicking the complex composition and hierarchical structure of human cementum, our biomimetic material exhibits a prodigious capacity to induce cementogenesis. The fabricated cementum provides us an innovative biomaterial for achieving cementum regeneration, and has the potential to be utilized as the fundamental structure to further constructing the cementum-periodontium-alveolar bone complex for future periodontal regeneration.

## Data Availability Statement

The raw data supporting the conclusions of this manuscript will be made available by the authors, without undue reservation, to any qualified researcher.

## Ethics Statement

The studies involving human participants were reviewed and approved by the Medical Ethics Committee of Hospital of Stomatology, Sun Yat-sen University. Written informed consent for participation was not required for this study in accordance with the national legislation and the institutional requirements. The animal study was reviewed and approved by Institutional Animal Care and Use Committee, Sun Yat-sen University.

## Author Contributions

WT, QX, and TY contributed substantially to the conception and design of the experiments. TY, YL, YH, LC, and CL conducted the experiments and wrote the manuscript. YL and YY conducted data analyses. QW revised and modified the draft.

## Conflict of Interest

The authors declare that the research was conducted in the absence of any commercial or financial relationships that could be construed as a potential conflict of interest.
