# Supplementary material for: The Construction of Biomimetic Cementum Through a Combination of Bioskiving and Fluorine-Containing Biomineralization
Source: Front Bioeng Biotechnol. 2020 Apr 24;8:341. doi: 10.3389/fbioe.2020.00341 (PMC7193115; doi:10.3389/fbioe.2020.00341)
Supplement: Supplementary file 1 [file Table_1.DOCX]

Supplementary Material


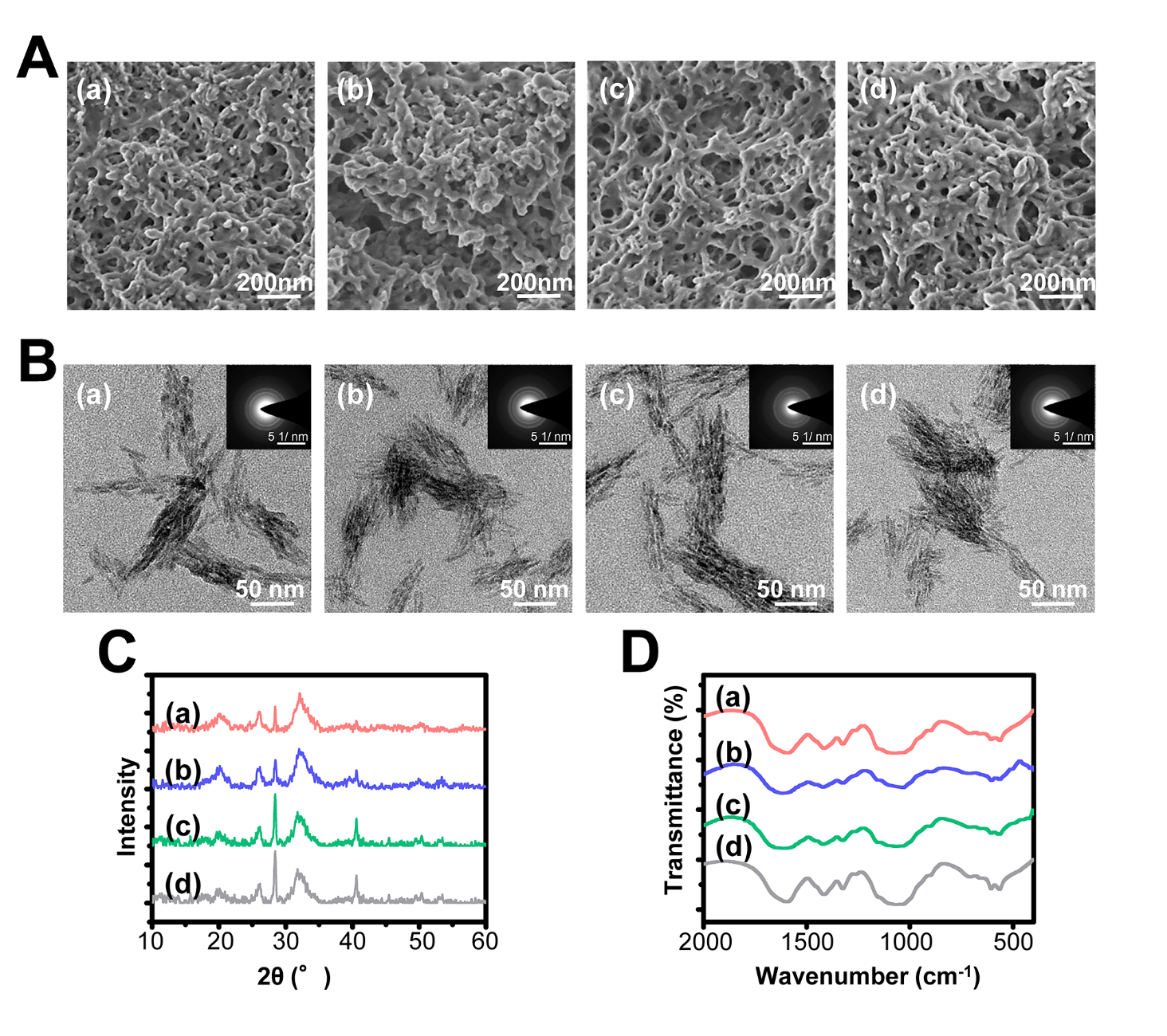


**Supplementary Figure 1.** Characterization of transformed FACP after 4 days. (A) SEM images, (B) TEM images and the corresponding SAED pattern, (C) XRD spectra and (D) FTIR spectra of FACP transformed after 4 days. In A-D, the initial fluorine concentration of FACP was (a) 0.625 mM, (b) 1.25 mM, (c) 2.5 mM and (d) 5 mM.


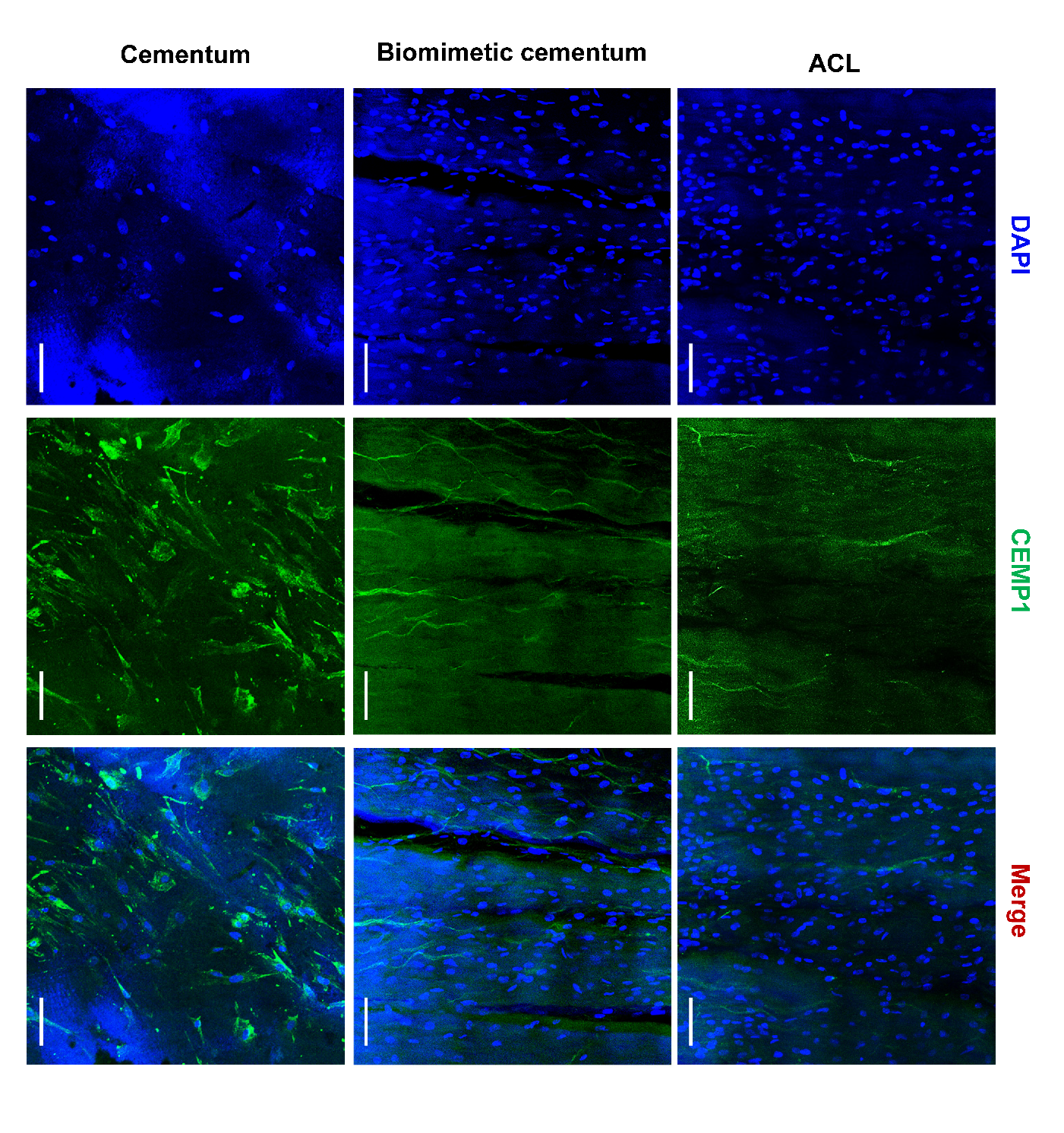


**Supplementary Figure 2.** The immunofluorescent image of CEMP1 protein expressed in PDLCs after 7 days. Blue fluorescence represents nuclei and green fluorescence represents CEMP1. Scale Bar: 100 μm.
